# Worse survival of invasive melanoma patients in men and “de novo” lesions^☆^^☆☆^

**DOI:** 10.1016/j.abd.2019.07.003

**Published:** 2020-01-23

**Authors:** Mara Huffenbaecher Giavina-Bianchi, Cyro Festa-Neto, Jose Antonio Sanches, Monica La Porte Teixeira, Bernadette Cunha Waldvogel

**Affiliations:** a Department of Dermatology, Faculdade de Medicina, Universidade de São Paulo, São Paulo, SP, Brazil; b Department of Data Information Analysis and Dissemination, Fundação Sistema Estadual de Análise de Dados, São Paulo, SP, Brazil

**Keywords:** Brazil, Epidemiology, Melanoma, Survival

## Abstract

**Background:**

The incidence and mortality of melanoma is increasing in many countries, including Brazil. Survival studies are still scarce in our country, but much needed to know and address this problem better.

**Objective:**

To analyze the disease-specific survival of patients with invasive melanoma and to correlate it with clinical and histopathological variables.

**Methods:**

Retrospective cohort analysis of 565 cases of invasive melanoma in a tertiary hospital with the objective of testing variables that could be associated with a worse prognosis, such as gender, phototype, thickness, histological type and presence of pre-existing clinical lesion at the site of the tumor.

**Results:**

The worst survival rates were significantly associated with thicker tumors (*p* < 0.001), male sex (*p* = 0.014), high phototype (*p* = 0.047), nodular melanoma (*p* = 0.024) and “de novo” lesions (*p* = 0.005). When all variables were adjusted for melanoma thickness, male patients (*p* = 0.011) and “de novo” melanomas (*p* = 0.025) remained associated with worse survival.

**Study limitations:**

Retrospective study of a single tertiary hospital.

**Conclusions:**

Although the causes are still unknown, melanoma-specific survival was statistically worse for males and for “de novo” melanomas even after adjustment of tumor thickness.

## Introduction

Melanoma is a skin cancer that kills more than all other skin cancers combined, even though it accounts for less than 5% of all cases.^1^ Recently, many new molecular and target therapies have been introduced for advanced stages melanomas,^2^, ^3^ but early detection and surgery are still the only reliable methods to increase survival. The incidence and mortality of melanoma are increasing around the world,^4^, ^5^, ^6^, ^7^, ^8^ and it is a public health problem in many countries. The incidence in the world rose from 11.8/100,000 inhabitants in the period 2003–2006 to 17.5 for the period 2011–2014.^9^, ^10^ In the USA, the number has jumped from less than 10 cases/100,000 inhabitants (1975) to about 25 in 2013.^5^ In Australia, there were 27 cases/100,000 inhabitants (1982) and 49 in 2012.^6^ A recently published study with Brazilian data showed that mean incidence in men increased from 2.52 to 4.84 and in women, from 1.33 to 3.22 cases/100,000 inhabitants in 13 years (2000–2013) in the country.^11^

In Australia, 10.2% of all cancers were diagnosed as melanomas, in USA, 4.5% and in Brazil, less than 2.3%. New melanoma cases were 13,283 in 2012 in Australia, 76,380 in 2013 in the USA and 5670 in 2012 in Brazil.^4^, ^5^, ^6^

There are few epidemiologic studies focusing on the survival rates of melanoma in Brazil. For this reason, we performed this project in Hospital das Clínicas–FMUSP, with the objective of analyzing the data of disease-specific survival of patients with invasive primary cutaneous melanoma followed in our service regarding: sex, skin phototype, Breslow Index, histological subtype and clinical presence of pre-existing clinical lesion at the tumor site.

## Methods

This work was approved by the Ethics Committee of Hospital das Clínicas–FMUSP (CAAE: 76591317.0.0000.0068). This study was designed to be a retrospective cohort analysis of 565 cases of invasive melanomas to test variables associated with poor prognosis. All data from the patients diagnosed with melanoma followed at Oncology Outpatient Clinic at Hospital das Clínicas da FMUSP, between January 1987 and May 2016, were reviewed and analyzed. Survival data were obtained through Fundação Sistema Estadual de Análise de Dados (Seade), which is a government agency of the São Paulo, that kindly shared with us the date and cause of death of our patients.

Inclusion criteria were: Invasive melanoma diagnosed between January 1987 and May 2016; Melanoma thickness (Breslow Index) measured and reported by the dermatopathologist.

A spreadsheet used by the physicians to follow the melanoma patients was the source of the information, as well as the medical records, when necessary. The data of the patients searched were: sex, age at diagnosis, phototype Fitzpatrick phototype, melanoma location, presence of pre-existing clinical lesion at the tumor site, vital status of the patient at the last known visit: alive or dead and, in the latter case, what was the cause of death according to 10^th^ International Code of Diseases (ICD-10). Patients who were followed for less than 3 months from the date of diagnosis or who did not present the date of the last query on the worksheet were considered as missing as to their vital status. Phototypes I, II and III were classified as low and, IV, V and VI, as high.

The data collected from the histological report were: Breslow Index, histological subtype, presence of ulceration and regression, mitosis (measured according to AJCC: American Joint Committee on Cancer recommendation) and sentinel lymph node status. For patients who had more than one primary invasive melanoma, we opted for the thicker tumor data for survival-specific analysis. The survival data from Fundação SEADE was obtained by linking the following information: name and date of birthday of the patient, mother's name and cause of death.

Survival analysis was used to evaluate the overall survival for melanoma patients. Survival curves were constructed using Kaplan–Meier method and compared by log-rank test. Mean and median times as well as standard error were calculated for overall survival of all patients and for each category of independent variables.

The Cox regression was used to calculate Hazard Ratios (HR) and the respective 95% Confidence Intervals for each of the independent variables. The final model was adjusted by all independent variables. Additionally, each one of the variables: sex, photoytpe and pre-existing or de novo clinical lesion was adjusted by Breslow Index. The level of significance was 5%. Analyzes were performed in statistical software SPSS v.18 for Windows and Stata v.11.

## Results

The spreadsheet of the at Oncology Outpatient Clinic at Hospital das Clínicas da FMUSP outpatient clinic used to store the data of patients with melanoma followed in the service had 906 patients from its beginning between January 1987 and May 2016. According to the criteria defined above, we excluded 173 cases of melanoma in situ and 177 that, although invasive, did not present the Breslow Index reported in the histopathological report. For this reason, our cohort contained 556 patients. As nine of them had two distinct primary invasive melanomas, this survey included 565 melanomas.

### Clinical and histopathological

To give an idea of the robustness of the study population, table 1 summarizes all the clinical and histopathological data computed. Briefly, we can say that our cohort was mostly female patients (61%); that the highest frequency of diagnosis occurred between 50 and 80 years (60%), with a mean age of 56.6 years. Regarding the Fitzpatrick phototype, type II was the most frequent (44%), followed by type III (29%). The most frequent sites of invasive melanomas were trunk with 37%, followed by limbs with 34%, head and neck, 16% and extremities (feet and hands), 12%.

**Table 1:** Clinical characteristics of patients and histological parameters with invasive primary cutaneous melanomas followed by Cutaneous Oncology Outpatient Clinic of Department of Dermatology at HC-FMUSP from 1987 to May 2016

Characteristics	*n* (%)
*Sex*	
Male	218 (39)
Female	338 (61)

*Age at diagnosis (years)*	
0–10	1 (0.2)
10–19	0 (0)
20–29	24 (4)
30–39	54 (10)
40–49	99 (18)
50–59	118 (21)
60–69	138 (25)
70–79	80 (14)
80–89	28 (5)
90–100	6 (1)
No information	8 (1)

*Phototype according to Fitzpatrick*	
I	22 (4)
II	247 (44)
III	159 (29)
IV	26 (5)
V	12 (2)
VI	0 (0)
No information	89 (16)

*Place*	
Head/Neck	90 (16)
Trunk	211 (37)
Members	196 (34)
Ends	68 (12)

*Breslow index*	
≤1.0	279 (51)
1.01–2.00	120 (21)
2.01–4.00	85 (15)
>4.0	81 (14)

*Ulceration*	
Present	60 (10)
Absent	450 (80)
No information	55 (10)

*Regression*	
Present	63 (11)
Absent	424 (75)
No information	78 (14)

*Histological type*	
Superficial extensive	262 (46)
Nodular	62 (11)
Malignant lentigo	45 (8)
Acral lentiginous	51 (9)
Not classified	6 (1)
No information	139 (25)

*Mitosis*	
Present	87 (15)
Absent	66 (12)
No information	412 (73)

*Sentinel lymphnode*	
Positive	24 (4)
Negative	89 (16)
Unrealized	31 (5)
No information	421 (75)

*Pre-existing lesion*	
Yes	199 (35)
No	285 (51)
Ignored	13 (2)
No information	68 (12)

The mean Breslow index for all melanomas was 2.01 mm. Melanomas ≤1.0 mm were 51% of the cases; 21% were between 1.01 and 2.0 mm; 15% between 2.01 and 4.0 mm and 14% with thickness >4.0 mm. Ulceration and regression were identified in 10% and 11% of tumors, respectively. Superficial superficial melanoma accounted for 46% of the cases; followed by nodular type, with 11%, acral lentiginous with 9% and lentigo malignant melanoma with 8%. Mitoses were present in 15% of melanomas, absent in 12% of them, and 73% of the reports did not specify this information. The sentinel lymph node survey showed that 16% were negative, 4% positive and in the other 80% had not been performed. Regarding the presence of pre-existing clinical lesion, 51% denied, 35% confirmed and 14% did not know how to report.

### Melanoma-specific survival

In this study 556 melanoma patients were included, of whom 521 (93.7%) had information on vital status (alive or dead). Of the patients with available information, 463 (88.9%) were alive and 58 (11.1%) were deaths. Survival analysis was considered in months, calculation of survival probabilities at 1 year, 2 year, 5 year and 10 year survival was performed using the Kaplan–Meier method. Mean median and standard error values were presented for the total number of patients and according to the variables: Breslow index, sex, phototype and type of lesion (pre-existing or de novo). The comparison of the survival curves showed statistically significant differences between the categories of the Breslow index (*p* < 0.001), sex (*p* = 0.014), phototype (*p* = 0.047) and type of injury (*p* = 0.005) (Table 2).

**Table 2:** Probability of overall survival according to Breslow index

	Average (SE) (months)	Median (months)	Probability of survival				Value of *p*^a^
			1 year	2 years	5 years	10 years	
*Global*	400.10 (23.40)	NA	99.6%	98.6%	95.7%	84.6%	
*Breslow index*							<0.001
≤1.00 mm	459.28 (27.34)	NA	100%	99.5%	98.7%	92.4%	
1.01–2.00 mm	252.32 (22.70)	NA	100%	100%	96.8%	91.7%	
2.01–4.00 mm	272.96 (20.43)	NA	100%	98.4%	93.6%	75.1%	
>4.00 mm	154.71 (14.74)	164.00	97.1%	93.7%	86.6%	58.2%	

*Sex*							0.014
Female	439.87 (27.89)	NA	99.3%	98.5%	95.7%	87.7%	
Male	216.11 (17.47)	194.00	100%	98.8%	95.7%	79.2%	

*Phototype*							0.047
High	419.73 (30.52)	NA	100%	99.4%	97.5%	90.8%	
Low	181.42 (22.43)	194.00	100%	100%	95.0%	62.4%	

*Type of lesion*							0.005
Pre-existing	425.69 (51.57)	NA	100%	98.6%	96.6%	95.3%	

*Again*	239.74 (14.69)	NA	99.2%	98.7%	94.5%	74.7%	

HR, Hazard Ratio; NA, not available.

a Log-rank test.

As shown in the table above, overall survival decreases over the years. From 99.6% in 1 year it goes to 84.6% in 10 years. The higher the Breslow Index, the lower the survival rates. The women had better survival than the men, as well as low phototypes compared to the high ones, and the presence of pre-existing clinical lesion at the site of the appearance of melanoma compared with those de novo melanomas.

In the evaluation of factors related to overall survival, unadjusted and adjusted Cox regression was used. Hazard Ratio (HR) values and their respective 95% Confidence Intervals were calculated and presented in table 3 . The Breslow index continued to be strongly significant, showing that thicker tumors, especially larger than 4 mm, have a worse survival than thinner ones (*p* = 0.001). Female patients maintained a better prognosis than men (*p* = 0.018). In this analysis, the high versus low phototype and the presence or absence of pre-existing clinical lesion were not associated with the survival rate.

**Table 3:** Overall survival in patients with melanoma using Cox regression

Breslow index (mm)	Deaths/Total (%)	HR not adjustaded (95%CI)	Value of *p*^a^	HR ajustado (95%CI)	Value of *p*^b^
*≤1.00*	17/257 (6.6)	1		1	
*1.01–2.00*	12/112 (10.7)	1.56 (0.66–3.70)	0.315	2.02 (0.71–5.78)	0.190
*2.01–4.0*	12/78 (15.4)	2.81 (0.91–5.20)	0.079	2.59 (0.87–7.71)	0.089
*>4.00*	17/74 (23.0)	5.13 (2.40–10.97)	<0.001	5.37 (1.96–14.76)	0.001

*Sex*					
Female	30/312 (9.6)	1		1	
Male	28/209 (13.4)	2.06 (1.15–3.71)	0.016	2.40 (1.17–4.96)	0.018

*Phototype*					
High	35/411 (8.5)	1		1	
Low	6/35 (17.1)	2.40 (0.98–5.86)	0.054	0.92 (0.33–2.56)	0.871

*Type of lesion*					
Pre-existing	16/180 (8.9)	1		1	
*Again*	41/261 (15.7)	2.67 (1.32–5.40)	0.006	2.05 (0.91–4.62)	0.082

HR, Hazard Ratio; 95%CI, 95% Confidence Interval.

a *p*-Value for unadjusted regression Cox model.

b *p*-Value adjusted by all variables using regression Cox model.

Next, we wanted to verify the Cox regression model by adjusting each of the independent variables by the Breslow index. The results are presented in table 4. Only the phototype variable did not result in a significant association when comparing the survival curves adjusted by Breslow Index (*p* = 0.426). Sex (*p* = 0.025) and type of lesion (*p* = 0.011) showed significant differences in the survival curves adjusted by the Breslow index. The women and melanomas that appeared on pre-existing clinical lesions remained with better survival when compared to men and de novo melanomas respectively.

**Table 4:** Overall survival in patients with melanoma adjusted by Breslow index, using Cox regression

	Deaths/Total (%)	HR not adjustaded (95%CI)	Value of *p*^a^	HR adjustaded (95%CI)	Value of *p*^b^
*Sex*					
Female	30/312 (9.6)	1		1	
Male	28/209 (13.4)	2.06 (1.15–3.71)	0.016	1.96 (1.09–3.53)	0.025

*Phototype*					
High	35/411 (8.5)	1		1	
Low	6/35 (17.1)	2.40 (0.98–5.86)	0.054	1.47 (0.57–3.80)	0.426

*Type of lesion*					
Pre-existing	16/180 (8.9)	1		1	

*Again*	41/261 (15.7)	2.67 (1.32–5.40)	0.006	2.52 (1.24–5.11)	0.011

HR, Hazard Ratio; 95%CI, 95% Confidence Interval.

a *p*-Value for unadjusted regression Cox model.

b *p*-Value adjusted by Breslow index using regression Cox model.

Histological subtypes of melanoma showed different survival curves with statistical significance (*p* = 0.024) (Table 5). The superficial spreading melanoma type presented the highest percentage of patients alive at the end of the study, differing from the rest of them.

**Table 5:** Disease-specific survival in patients with melanoma according to histological type

Variable	Total	Alive *n* (%)	Deaths *n* (%)	Average (SE) (months)	Median (months)	Value of *p*^a^
*Disease-specific survival*	371	343 (92.5)	28 (7.5)	394.23 (30.38)	NA	

*Histological type*						0.024
I (MES)	231	221 (95.7)	10 (4.3)	451.59 (38.46)	NA	
II (NOD)	56	48 (85.7)	8 (14.3)	236.56 (29.63)	203.00	
III (LMM)	40	36 (90.0)	4 (10.0)	195.03 (30.14)	NA	
IV (MLA)	44	38 (86.4)	6 (13.6)	166.16 (13.50)	194.00	

SE, standard error; NA, not available, SSM, superficial spreading melanoma; NOD, nodular melanoma; LMM, Lentigo Maligno Melanoma; ALM, Acrolentiginous Melanoma.

a *p*-Value for unadjusted Log-rank test.

Table 6 analyzes the specific-disease survival of patients with melanoma according to histological type and Breslow Index. In univariate form, with unadjusted HR, we can see that histological melanoma types II (nodular) and IV (acral lentiginous), as well as Breslow Index, presented a significant difference in relation to superficial spreading type. In the multivariate analyzes, with adjusted HR, there was no correlation between histological type and survival.

**Table 6:** Univariated and Multiple Cox Regression for disease-specific survival in patients’ melanoma according to histological type

Variable	HR not adjustaded (95% CI)	HR adjustaded (95% CI)	Value of *p*^a^
*Histological type*			
I (MES)	1.00	1.00	
II (NOD)	2.95 (1.16–7.50)	1.21 (0.32–4.58)	0.774
III (LMM)	3.02 (0.94–9.64)	3.18 (0.99–10.18)	0.051
IV (MLA)	3.49 (1.26–9.68)	2.54 (0.87–7.43)	0.088
Breslow	1.18 (1.07–1.29)	1.18 (1.02–1.36)	0.027

HR, Hazard Ratio; 95%CI, 95% confidence interval; SSM, superficial spreading melanoma; NOD, nodular melanoma; LMM, Lentigo Maligno Melanoma; ALM, Acrolentiginous Melanoma.

a *p*-Value adjusted for Breslow using Cox regression test.

## Discussion

The results shown in table 1 confirm the robustness of our cohort and have many resemblances to other published studies in Brazil. Two of them demonstrated a higher incidence of melanoma in women, as ours.^10^, ^11^ Another article revealed a higher frequency of melanoma on trunk and superficial spreading type, similar to our study. However, a higher prevalence of intermediate and thick tumors was observed, which is different from our casuistic, composed most by thin tumors.^12^

The mortality rate of melanoma has also increased over the years. Brazil had 0.06 in 1979 and 0.13 per 100,000 inhabitants in 2014.^4^ In Australia, one of the countries with the highest incidence of this pathology in the world, was 3.3 in 1968 and 6.2 per 100,000 inhabitants in 2013.^6^ Recently published, a Brazilian study using a database with more than 28,000 individuals revealed an increase in mortality from 0.85 to 0.9/100,000 inhabitants in men and a decrease from 0.56 to 0.53/100,000 inhabitants in women between 2000 and 2014.^11^

The 5 year survival rate of melanoma in Australia, USA, and Netherlands is respectively: 90%, 91.5% and 86%. In our study, it was 95.7% in 5 years, remembering that we included only invasive cases, which, in theory, should worsen our survival statistics. It should also be taken into account that we lost the follow-up of a part of the patients, due to lack of consultations or lack of data, such as Breslow Index, which was not determined in 17% of the cases, and deaths occurred outside the state of São Paulo, which could bring some bias to the study. Another limitation of the project is that it is retrospective and has been conducted in a single tertiary hospital, which may not necessarily reflect the conditions of the country as a whole, such as the rates of the countries mentioned above.^4^, ^5^, ^6^, ^13^, ^14^

The Breslow index is an accepted parameter for staging melanomas and predicting melanoma-specific survival. Individuals with thinner tumors have better survival, as can be seen in our disease-specific survival analysis and in other publications.^15^, ^16^ Our data show that only 7.6% of tumors ≤1.0 mm died of melanoma in 10 years compared to 41.8% of those thicker than 4.0 mm in the same period. The chance of death of a melanoma patient with Breslow >4.0 mm is 5.37× greater than an individual with melanomas with Breslow ≤1.0 mm. It is worth noting that, in any case, even thin tumors (≤1.0 mm) led to death in 7.6% of the cases (Table 2).

Survival in our sample was different according to sex. Men had a significantly worse prognosis than women. Being a man increased the chance of death from melanoma (Table 3). In the US, this is also true: there are 4.1 deaths in men and 1.7 in women per 100.00 people^5^, ^15^; in Japan, survival at 140 months was 70.6% in women and 60% in men.^8^ A study in England also reported that being a man had a negative impact on survival.^17^ In our study, even after adjustment for the Breslow index, which alone is the most important parameter in the survival of the melanoma patient, the male patients continued to have a worse prognosis (Table 4), which had not been previously tested. The reasons for this remain speculative.

Regarding phototype/race and survival, we found a lower survival rate in patients with Fitzpatrick high phototypes (IV, V and VI), but this difference was not maintained after adjustment for the Breslow index (Table 4). One explanation for this may be that patients with higher phototypes may be diagnosed later, leading to a worse prognosis than those with lower phototypes. Socioeconomic problems could delay access to the health care system and/or the belief that skin cancer is a problem only for patients with low phototype may be contributing to this delay. Most studies evaluate race. One article found that black people have a lower survival rate, but did not adjust for thickness.^7^ Another, showed that non-Hispanics blacks have a better survival rate than non-Hispanic whites, which seems contrary to our findings, but there was also no adjustment for the Breslow index.^18^

Another interesting finding is that de novo melanomas are more aggressive than those that develop on pre-existing clinical lesions (Table 2, Table 4), which was corroborated in another study.^19^ We might speculate that mutations that lead to the onset of de novo melanoma could cause a more aggressive disease than other mutations that occur in pre-existing lesions.

Histological types have different probabilties of survival rates as well. In our study, superficial spreading melanoma obtained higher probability of survival than the other types (Table 5). A Japanese study revealed that nodular melanoma had the worst prognosis.^8^ However, this may occur not because of the subtype of melanoma itself but because, usually, at diagnosis, the nodular melanoma is thicker and the superficial spreading melanoma is thinner. This hypothesis can be corroborated by our resullts, in which we found that, once the Breslow index was adjusted for all histological types of melanoma, there was no statistical difference between them.

Survival data are the major differential of our study. For few years now, our hospital has an agreement with the SEADE Foundation, a government agency that lets us know the cause and date of the patients’ deaths. Surely this is essential to obtain the curves of mortality and survivals presented here and also enable us to plan public health policies to better treat and prevent deaths from melanoma.

## Conclusion

Survival analyzes of patients with invasive melanoma from our retrospective cohort showed association with tumor thickness, sex, and presence or absence of pre-existing clinical lesion. Future studies may verify if there is an association between survival and histological subtype or phototype of the patient, as our results suggest.

## Financial support

Own and FAPESP project number: 2017/20928-9.

## Authors’ contributions

Mara Huffenbaecher Giavina-Bianchi: Approval of the final version of the manuscript; conception and planning of the study; elaboration and writing of the manuscript; obtaining, analysis, and interpretation of the data; critical review of the literature; critical review of the manuscript.

Cyro Festa Neto: Approval of the final version of the manuscript; conception and planning of the study; elaboration and writing of the manuscript; effective participation in research orientation; critical review of the manuscript.

Jose Antonio Sanches: Approval of the final version of the manuscript; conception and planning of the study; elaboration and writing of the manuscript; effective participation in research orientation; critical review of the manuscript.

Monica La Porte Teixeira: Conception and planning of the study; obtaining, analysis, and interpretation of the data.

Bernadette Cunha Waldvogel: Conception and planning of the study; obtaining, analysis, and interpretation of the data.

## Conflicts of interest

None declared.
